# Quantitative analysis of gait parameters in Parkinson’s disease and the clinical significance

**DOI:** 10.3389/fneur.2025.1527020

**Published:** 2025-08-20

**Authors:** Wenchao Yin, Hong Gao, Beichen Liang, Ruichen Liu, Yue Liu, Chenxin Shen, Xiaohui Niu, Cui Wang

**Affiliations:** ^1^Department of Neurology, Central Hospital of Dalian University of Technology, Dalian, China; ^2^School of Science, Dalian Maritime University, Dalian, China; ^3^School of Computational Science and Engineering, Georgia Institute of Technology, Atlanta, GA, United States; ^4^Key Laboratory of Intelligent Control and Optimization for Industrial Equipment of Ministry of Education and the School of Control Science and Engineering, Dalian University of Technology, Dalian, China; ^5^Department of Neurology, The Fifth People’s Hospital of Shenyang, Shenyang, China

**Keywords:** Parkinson’s disease, gait disorder, levodopa, MDS-UPDRS III, machine learning

## Abstract

**Background:**

Gait disorder is one of the clinical manifestations of Parkinson’s disease (PD). Investigating the characteristics of gait disorder in patients with PD and the changes in gait before and after taking levodopa is crucial for the recognition, diagnosis and treatment of gait disorders in PD patients.

**Methods:**

In this study, we measured the gait parameters of 20 patients with PD and 17 healthy controls and analyzed the changes of gait parameters of these patients before and after taking levodopa. We also used gait parameters as input features and MDS-UPDRS III score (which was further subdivided into tremor and non-tremor part score) as output labels to train machine learning regression models.

**Results:**

We found that except for cadence and stride time, most gait parameters of PD patients, including plantar dorsiflexion angle, plantar flexion angle, stride length, velocity were all smaller than those of the healthy controls. Moreover, the severity of gait disorders correlated with the severity of motor symptoms. After taking levodopa, the stride length, velocity and cadence were increased, but stride time was decreased. We also found that the trained machine learning model could explain and predict the MDS-UPDRS III score and non-tremor part score, and the non-tremor part score was better than the MDS-UPDRS III score.

**Conclusion:**

Our gait assessment work can help clinicians recognize gait disorder in PD patients and predict the severity of clinical symptoms.

## 1 Introduction

Parkinson’s disease (PD) is one of the most common neurodegenerative diseases affecting nearly 1‰ of the global population (1). The main pathological features of PD are degeneration and loss of Dopamine (DA) neurons in Substantia Nigra (SN) and formation of Lewy bodies in the remaining DA neurons, leading to a reduced amount of DA in the brain (2). At present, PD cannot be cured, the treatment mainly focuses on the DA replacement strategy to relieve symptoms (3).

The typical motor symptoms of PD include tremor at rest, bradykinesia, rigidity, gait and posture disorders. Gait disorder is one of the most common motor symptoms in PD. It mainly shows slow walking speed, short stride length and frozen gait, they can lead to unstable in postural and fall, which is one of the important reasons for the decline in quality of life in PD (4, 5). Therefore, the recognition and monitoring of gait disorder in PD is of great significance for the treatment and prognosis of PD patients.

At present, the clinical assessment of gait includes some scales and tests, including Section III of the modified movement disorder society version of the unified Parkinson’s disease rating scale (MDS-UPDRS III), Hoehn and Yahr (H&Y) stage, Freezing of Gait Questionnaire (FOG-Q), Timed Up and Go Test, etc. (6). But the results are semi-quantitative and subjective, the accuracy and objectivity cannot be guaranteed.

Besides clinical assessments, the main research on gait parameters of PD is optical motion capture technology. Multi-camera motion capture system is considered to be the golden standard in clinical gait analysis owing to the high accuracy (7). However, the equipment is expensive, occupies a large area, requires complex setting stages, and most medical institutions cannot support such tests. A few researches use mobile phone software to collect data, but the technology is not mature at present, and the accuracy and reliability cannot be guaranteed, thus, it is not widely available for the time being. Several validation studies compare wearable sensor with optical motor capture system, confirming that wearable sensor is a simple and reliable means to assess gait parameters in PD patients (8, 9). Wearable sensor is rapidly replacing sophisticated camera-based motion capture system because of its convenience and portability, allowing clinicians to assess gait objectively and quantitatively outside laboratory (10).

The existing research results showed that the walking speed and stride length of PD patients were decreased compared with healthy people (11, 12). However, these studies were conducted under the state of drug on (ON) or drug off (OFF), the differences between the two states have not been fully investigated, which brings limitations to the results. Therefore, the analysis of ON and OFF states in PD patients is needed in further study (12). Recently, some researchers have studied the effects of DA drugs on gait parameters, but the conclusions are inconsistent. For example, Curtze et al. (13) found that the velocity and stride length were increased in ON state. While Schlenstedt et al. (14) found that there was no significant difference in gait parameters such as step length and step velocity between ON and OFF states. In addition, it is still not clear how the quantitative assessment of gait compares to the assessment in the clinic and whether they should replace or merely complement current gold standard measures such as UPDRS (10).

On the basis of previous studies, we detected gait parameters of PD patients by placing sensors on the patients’ feet. We found that the main differences between PD patients and healthy people were slow velocity and short stride length, these conclusions were consistent with the results of other research groups (10, 15). Further, in order to evaluate its clinical significance, we explored whether there was a correlation between gait parameters and the scores of MDS-UPDRS III, and explored the specific changes of gait parameters caused by DA. At the same time, we constructed three predictive models to explore the predictive value of gait parameters, gait parameters were used as input features, MDS-UPDRS III score, tremor part score and non-tremor part score were used as output labels.

## 2 Methods

### 2.1 Participants

In this study, 20 participants with idiopathic PD were enrolled. All patients met the 2015 Movement Disorder Society (MDS) diagnosis criteria for primary PD (16), including 8 males and 12 females, the patients were between stage 1 and 3 on the H&Y scale (namely early-to-middle stage patients). At the same time, 17 age- and gender-matched healthy participants were recruited as the healthy control (HC) group, including 9 males and 8 females. Participants from both groups were between 45 and 85 years old, and all of them could walk 10 meters or more without assistance. The participants with significant systemic diseases (such as musculoskeletal, cerebrovascular, cardiovascular, respiratory) and other neurological diseases or uncorrected visual disturbances or ailments that might modify the gait patterns were actively selected and excluded from the study. This study was approved by the Ethics Committee of Central Hospital of Dalian University of Technology (Reference No. YN2022-039-57). All the study participants signed the informed consent document prior to the study participation. The study was performed according to the guidelines of the declaration of Helsinki.

### 2.2 Clinical assessment

Demographic information was collected, including age, gender, height, weight and disease duration. General neurological examination and clinical scales assessment were performed and recorded by two experienced neurologists in movement disorders. Dopaminergic therapies were recorded, levodopa equivalent morning dose was calculated (levodopa equivalent dose (LED) is calculated as levodopa dose + levodopa dose × 1/3 (if on entacapone) + piribedil (mg) + pramipexole (mg) × 100 + selegiline (mg) × 10 + rasagiline (mg) × 100 + amantadine (mg) + controlled-release levodopa (mg) × 0.75) (17).

The severity of motor symptoms in PD patients were assessed using the H&Y staging scale and MDS-UPDRS III scale. H&Y staging scale ranges from 0 (no symptoms) to 5 (wheelchair bound or bedridden unless aided), MDS-UPDRS III: each item ranges from 0 to 4 (0 = normal, 1 = slight, 2 = mild, 3 = moderate, 4 = severe), there are 33 items in total, and the score of each item are added up to get a total score, ranging from 0 to 132 points.

### 2.3 Gait assessment

**Figure 1:**
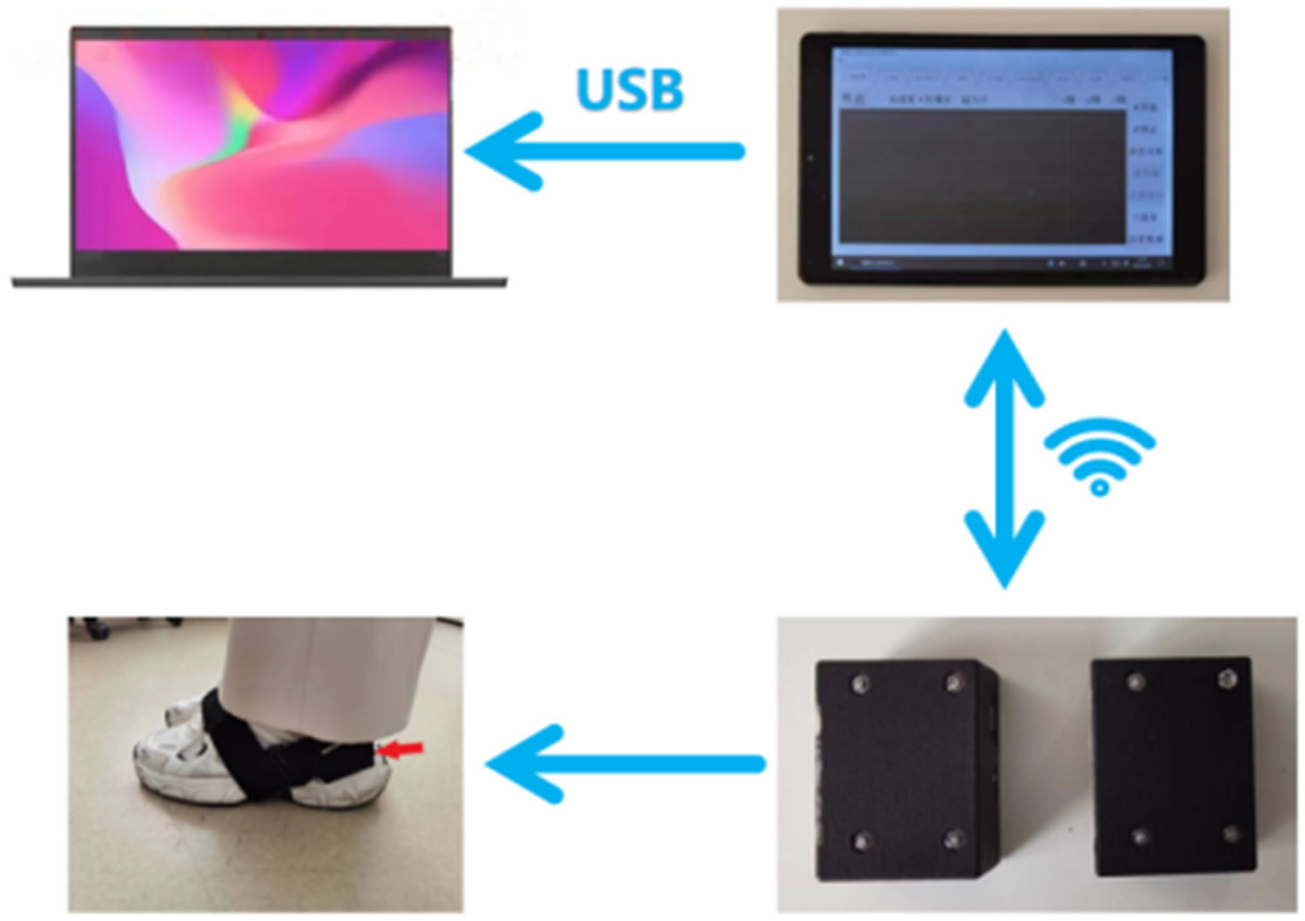
The structure of the gait data acquisition system.

**Figure 2:**
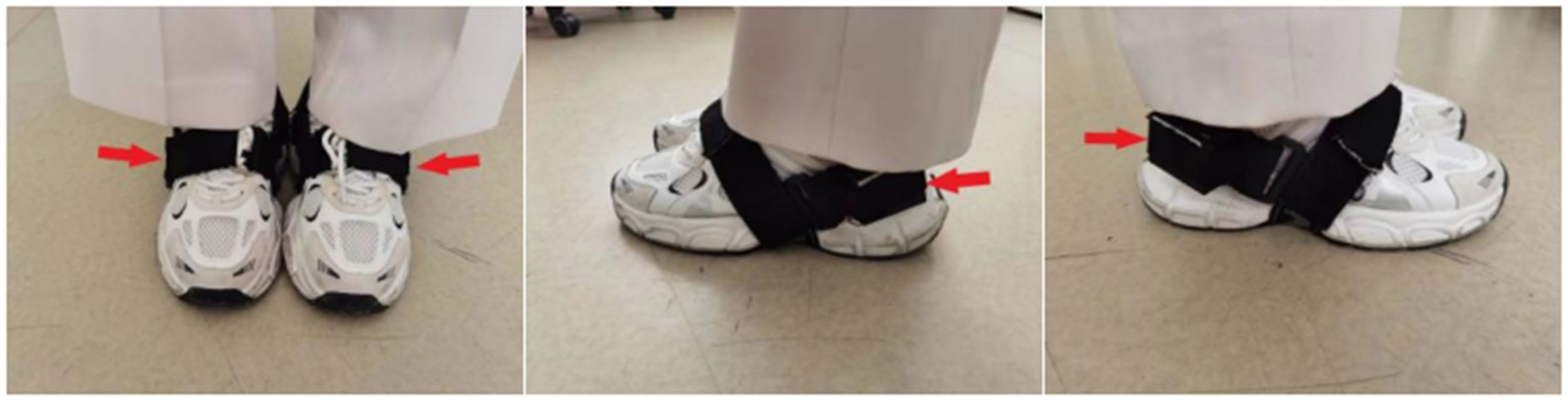
The placements of sensor nodes.

The gait data acquisition system consists of a self-developed handheld device and two sensor nodes (sampling frequency is 200 Hz) (Figure 1). Each sensor node is integrated with a microcontroller, a WIFI wireless communication module, a 450 mAh lithium battery and an inertial measurement unit (IMU) sensor (specific parameters are shown in Table 1), all those are packaged in a customized 3D printing box. The size of sensor node is 4.6 × 3.4 × 2.2 cm and the weight is 42 g. Each sensor node is attached to the lateral ankle of each foot (Figure 2). The working state of the sensor nodes is controlled by the handheld device. As “Start” is clicked, the two sensor nodes will start to collect data synchronously. The participant will stand for 3–5 s firstly (the data is used for correction at this time) and then start to walk. When the “Stop” command is sent to the sensor nodes, both nodes will be suspended, the collected data will be uploaded through wireless network and gait analysis will be performed.

**Table 1:** The specific parameters of sensor node.

Unit	Gyroscope	Accelerometer
Dimensions	3 axis	3 axis
Sensitivity (/LSB)	0.04 deg./s	0.833 mg
Dynamic range	±1,000 deg./s	±18 g
Bandwidth(kHz)	330	330
Alignment error (deg)	0.05	0.2

LSB, Least Significant Bit; kHz, kilohertz; deg, degree.

**Figure 3:**
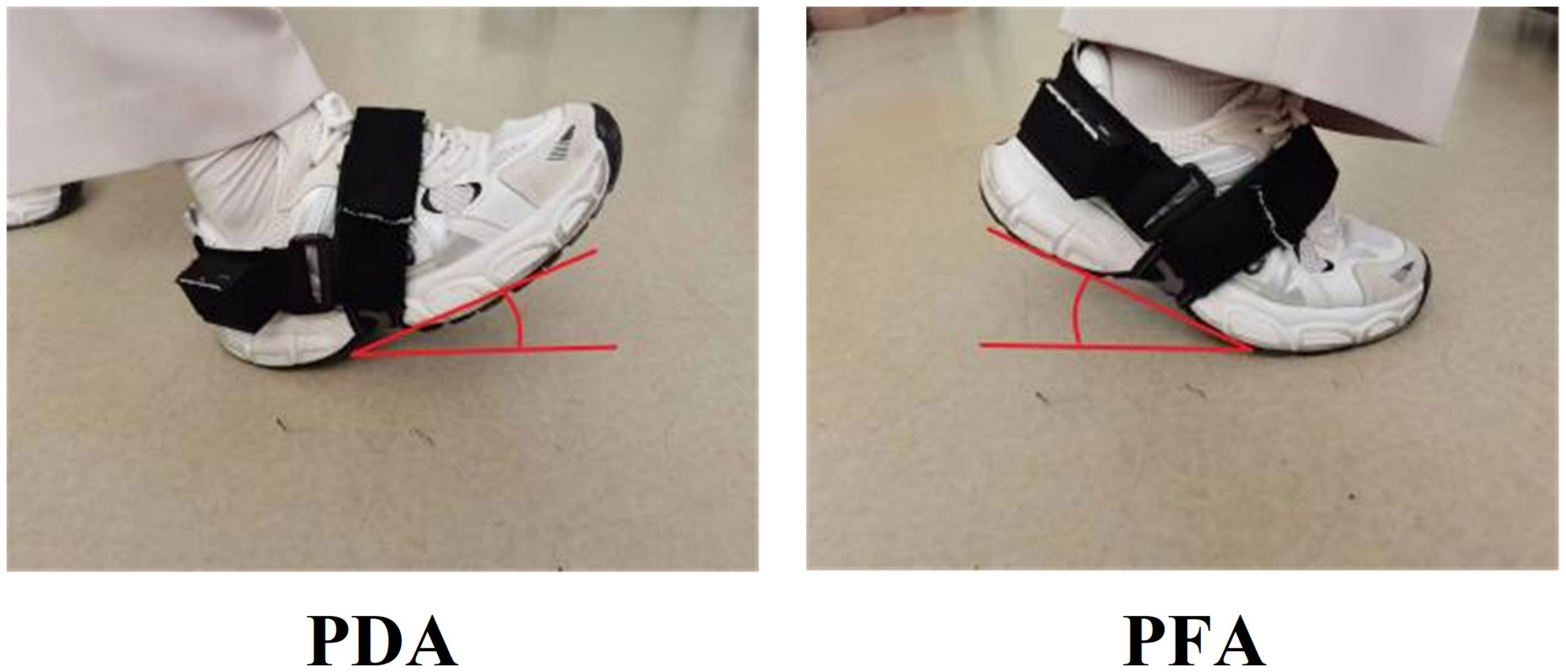
The definition of PDA and PFA.

Proprietary data acquisition technology is used to preprocess the original data and extract gait variables, calculate plantar dorsiflexion angle (PDA), plantar flexion angle (PFA), stride length, foot clearance, velocity, cadence and stride time (specific definitions are shown in Table 2 and Figure 3). The accuracy of the gait data acquisition system have been validated in previous studies (18), compared to the gold standard (optical motion capture), the position estimation error is less than 1% with regard to three dimensional motion.

**Table 2:** Specific definitions of gait parameters in this study.

Gait parameters	Definition
Plantar dorsiflexion angle (PDA) (°)	The absolute value of the angle between the foot and the ground at heel-strike moment.
Plantar flexion angle (PFA) (°)	The absolute value of the angle between the foot and the ground at toe-off moment.
Stride Length (m)	Distance between two consecutive heel-strikes, that is, the distance between the landing points of the same feet. Generally speaking, the stride length of the left and right feet can be calculated separately.
Foot clearance (m)	The maximal foot height during swing phase.
Velocity (m/s)	Calculated by dividing stride length by stride time.
Cadence (steps/min)	Steps per minute.
Stride time (s)	Duration between two heel strikes of the same foot.

The gait acquisition was performed in an obstacle free and flat environment. The participants were asked to walk independently through a 10-meter-long sidewalk at self-selected comfortable speed, in order to assess gait parameters objectively. There were no repeating instructions during the walk to avoid auditory cues. The first and last steps were excluded. Adequate protective measures were taken during the walking to ensure that the participants would not fall down suddenly.

### 2.4 Study procedure

PD patients were evaluated at both OFF state and ON state. In the OFF state, PD patients were fasting overnight, and at least 72 h without using dopamine agonists and at least 12 h without using any anti-Parkinson drugs. MDS-UPDRS III was scored and gait was assessed by two experienced neurologists in movement disorders. After the OFF test, they were re-tested in the ON state by the same neurologists, which was 2 h after 1.5 times of the regular morning levodopa dose or levodopa/benserazide (Modopar) 100/25–200/50 mg in the drug-naive patients. Healthy controls completed the same gait assessment but did not receive levodopa.

### 2.5 Modeling

In this study, we used gait parameters as input features and scale scores as output labels to train machine learning regression models. Specifically, we employed linear regression (implemented via Scikit-learn) as the modeling algorithm, which is suitable for small-sample regression tasks due to its simplicity and interpretability. To account for scale differences among features and improve model stability, all input features were standardized using z-score transformation prior to model training. The model was trained using default hyperparameters (regularization strength C = 1.0, solver = ‘liblinear’).

To further investigate the predictive value of gait parameters for the tremor and non-tremor components of the patient’s score, we divided the output labels into three categories: (1) MDS-UPDRS III score, (2) Tremor part score (i.e., items 3.15 + 3.16 + 3.17 + 3.18), and (3) Non-tremor part score (i.e., MDS-UPDRS III score minus tremor part score). A total of three prediction models were constructed.

Leave-one-out cross-validation was used to evaluate the performance of each model to assess the generalization ability and prediction accuracy of the model. Leave-one-out cross-validation is an effective method for evaluating the performance of regression models on a given data set. It does this by using one sample as the validation set and the remaining samples as the training set in each iteration, repeating this process until each sample has been used as the validation set, and finally averaging all the validation results to evaluate the performance of the model.

During the evaluation, the following metrics were used: R-squared (R^2^), Mean Absolute Error (MAE), and Mean Absolute Percentage Error (MAPE). R^2^ measures the goodness of fit of the model to the data, with a range from 0 to 1, and the closer it is to 1, the better the model fits the data. MAE measures the average absolute error between the predicted value and the true value, and the smaller values correspond to higher accuracy of the model. MAPE, on the other hand, is the average absolute percentage error expressed as a percentage, which can better assess the relative error between the predicted value and the true value, and is often used to understand the prediction accuracy of the model in different ranges.

### 2.6 Statistical analysis

The statistical analysis was carried out using SPSS 26.0 (IBM, Armonk, NY, United States) in our study. The Shapiro–Wilk test was used to check the normality of the data. Continuous variables with normal distributions were presented as means ± standard deviations ($\bar{x}\pm s$), the Independent Samples *t*-Test was used to analyze the differences between continuous variables. Continuous variables with non-normal distributions were presented as medians and interquartile distances [*M* (*P_25_, P_75_*)], and the Mann–Whitney *U* Test was used to analyze the differences. The Chi-square test was used to compare categorical variables. The Paired Samples *t*-Test or Wilcoxon Matched-Pairs Signed-Rank Test was used to analyze the changes between OFF and ON states. The Pearson correlation (*r*) or Spearman’s rank correlation (*r_s_*) was used to analyze the correlations between MDS-UPDRS III score and gait parameters. The statistically significant difference was considered *p* < 0.05 in two-tailed tests. In this study, machine learning regression models were trained and scatter plots were generated using Python 3.8 with sklearn and matplotlib.

## 3 Results

### 3.1 Demographic and clinical characteristics

The demographic information of PD group and healthy control group was shown in the first four lines of Table 3. We collected the data of gender, age, height and weight of 20 PD patients and 17 healthy controls. There was no statistically significant difference in their demographic characteristics, and *p* > 0.05 for all items was in line with the principle of object selection.

**Table 3:** Demographic and clinical characteristics in patients with PD and healthy controls.

Variable	PD (*n* = 20)	HC (*n* = 17)	*P* value
Gender (male/female)	8/12	9/8	0.517
Age (years)	69.15 ± 9.56	64.88 ± 3.62	0.077
Height (cm)	164.85 ± 7.76	166.65 ± 6.61	0.458
Weight (kg)	66.95 ± 8.47	67.18 ± 7.69	0.933
Disease duration (years)	1–8	NA	
MMSE (score)	20–30	NA	
H&Y stage	1–3	NA	
MDS-UPDRS III (OFF) (score)	36.55 ± 15.31	NA	

PD, Parkinson’s disease; HC, healthy control; MMSE, Mini-Mental State Examination; H&Y, Hoehn and Yahr; MDS-UPDRS III, section III of the modified movement disorder society version of the unified Parkinson’s disease rating scale; NA, not applicable; cm, centimeter; kg, kilogram; OFF, OFF state.

We collected the disease duration, H&Y stage and MDS-UPDRS III score in the OFF stage of PD patients; MMSE score in the ON stage of PD patients. The disease duration in PD group ranged from 1 to 8 years (median was 2.5 years), MMSE score ranged from 20 to 30 points (median was 27 points), H&Y stage ranged from 1 to 3, the mean MDS-UPDRS III score (OFF) was 36.55 ± 15.31, as shown in Table 3.

### 3.2 Comparison of gait parameters between PD patients and healthy controls

**Figure 4:**
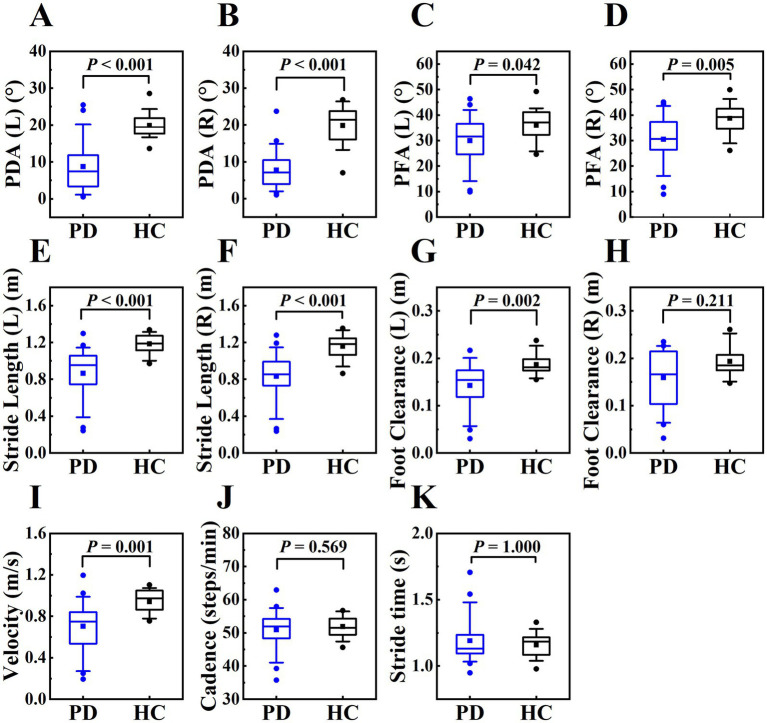
The boxplot of gait parameters between PD patients and healthy controls, including PDA (left, right) (plots **A** and **B**), PFA (left, right) (plots **C** and **D**), stride length (left, right) (plots **E** and **F**), foot clearance (left, right) (plots **G** and **H**), velocity (plot **I**), cadence (plot **J**), stride time (panel **K**). The box represents data from 25th to 75th percentile, the horizontal line inside the box represents the median, the solid box inside the box represents the mean, lower and upper error lines represent the 10th and 90th percentile respectively, filled circles represent the outliers.

The comparisons of gait parameters between the PD patients (OFF state) and the healthy controls are shown in Table 4 and Figure 4. Various gait parameters were significantly lower in PD patients than in healthy controls, including PDA (left, right), PFA (left, right), stride length (left, right), foot clearance (left), velocity (*p* < 0.05), the *p* values were lower than or equal to 0.001 in PDA, stride length and velocity among the above parameters, which indicated that PD patients tended to have a flat-footed gait pattern, slow velocity and short stride length when walking. We speculated that the statistically significant difference in foot clearance between left and right was due to the different statistical methods we used, the Independent Samples *t*-Test was used to analyze the differences between left foot, while and the Mann–Whitney *U* Test was used to analyze the differences between right foot, which leaded to the decrease of efficiency.

**Table 4:** Comparison of gait parameters between PD patients and healthy controls.

Gait parameters	PD (*n* = 20)	HC (*n* = 17)	*p* value
PDA (L) (°)	7.45 (3.26, 12.26)	19.91 ± 3.50	**< 0.001**
PDA (R) (°)	7.07 (3.73, 10.55)	19.85 ± 5.38	**< 0.001**
PFA (L) (°)	29.94 ± 9.98	36.02 ± 6.93	**0.042**
PFA (R) (°)	30.50 ± 9.73	38.71 ± 6.34	**0.005**
Stride Length (L) (m)	0.87 ± 0.28	1.18 ± 0.11	**< 0.001**
Stride Length (R) (m)	0.83 ± 0.27	1.16 ± 0.14	**< 0.001**
Foot Clearance (L) (m)	0.14 ± 0.05	0.19 ± 0.02	**0.002**
Foot Clearance (R) (m)	0.17 (0.10, 0.21)	0.19 ± 0.04	0.211
Velocity (m/s)	0.70 ± 0.26	0.94 ± 0.11	**0.001**
Cadence (steps/min)	50.92 ± 6.31	51.91 ± 3.43	0.569
Stride time (s)	1.13 (1.09, 1.24)	1.16 ± 0.10	1.000

PDA, plantar dorsiflexion angle; PFA, plantar flexion angle; L, left foot; R, right foot; °, degree; m, meter; min, minute; s, second; m/s, meters per second; steps/min, steps per minute. *p* values in bold indicate statistical significance (*p* < 0.05). *p* value < 0.05 should be shown in bold.

### 3.3 Correlation between MDS-UPDRS III score and gait parameters in PD patients

**Figure 5:**
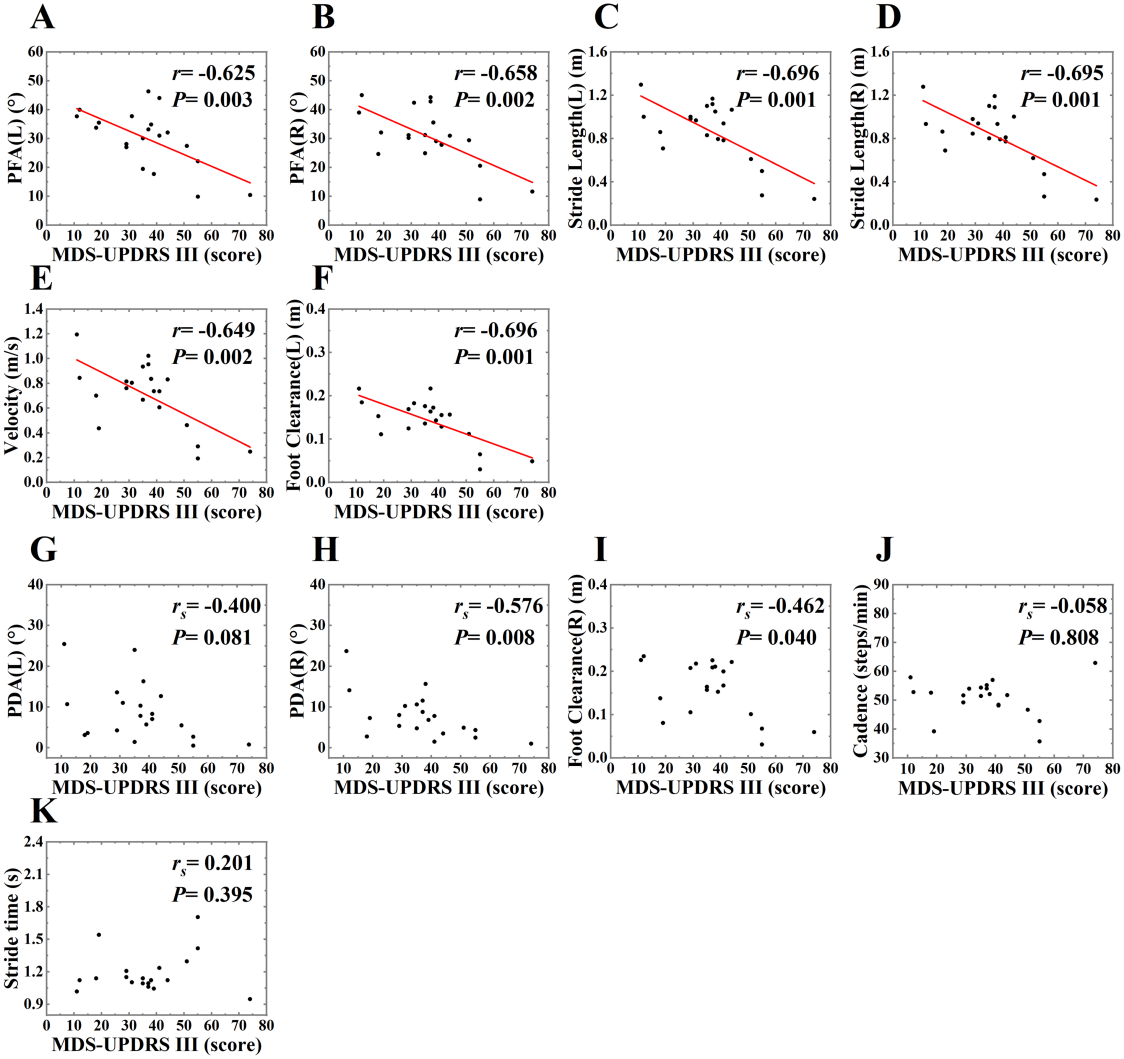
The scatter plot of correlations between MDS-UPDRS III score and gait parameters in PD patients in OFF state. The correlations between MDS-UPDRS III score and PFA (left, right), stride length (left, right), velocity, foot clearance (left) were shown on plots **(A–F)**, *r* on plots represented for Pearson correlation coefficient, *P* on plots represented for *p* value. We used the least squares method to provide the fitted curve due to the linear correlation, red line indicated line of best fit. The correlations between MDS-UPDRS III score and PDA (right), foot clearance (right) were shown on plots **(H,I)**, we only presented the scatter plots due to Spearman’s rank correlation, and *r_s_* on plots represented for Spearman’s rank correlation coefficient, *P* on plots represented for *p* value. There was no correlation between MDS-UPDRS III score and PDA (left), cadence, stride time, so we only presented the scatter plots, *r_s_* and *p* value on plots **(G, J,K)**.

In this study, we analyzed the correlation between gait parameters and MDS-UPDRS III score in PD patients in OFF state. MDS-UPDRS III score was negatively correlated with PDA (right), PFA (left, right), stride length (left, right), foot clearance (left, right) and velocity. These correlations indicated that the increased severity of motor symptoms was associated with the more obvious of the gait disorders, such as the flat-footed gait pattern, slow velocity, short stride length and low foot height. The PFA had a stronger correlation with the severity of motor symptoms compared with PDA. (Table 5 and Figure 5).

**Table 5:** Correlation between MDS-UPDRS III score and gait parameters in PD patients in OFF state.

Gait parameters	MDS-UPDRS III score (OFF)	
	Correlation coefficient (*r/r_s_*)	*P* value
PDA (L) (°)	−0.400	0.081
PDA (R) (°)	−0.576	**0.008**
PFA (L) (°)	−0.625	**0.003**
PFA (R) (°)	−0.658	**0.002**
Stride length (L) (m)	−0.696	**0.001**
Stride length (R) (m)	−0.695	**0.001**
Foot clearance (L) (m)	−0.696	**0.001**
Foot clearance (R) (m)	−0.462	**0.040**
Velocity (m/s)	−0.649	**0.002**
Cadence (steps/min)	−0.058	0.808
Stride time (s)	0.201	0.395

*r*, Pearson correlation coefficient. *r*_s_, Spearman’s rank correlation coefficient, *p* values in bold indicate statistical significance (*p* < 0.05). *p* value < 0.05 should be shown in bold.

### 3.4 Comparison of gait parameters between the OFF and ON states in PD patients

**Figure 6:**
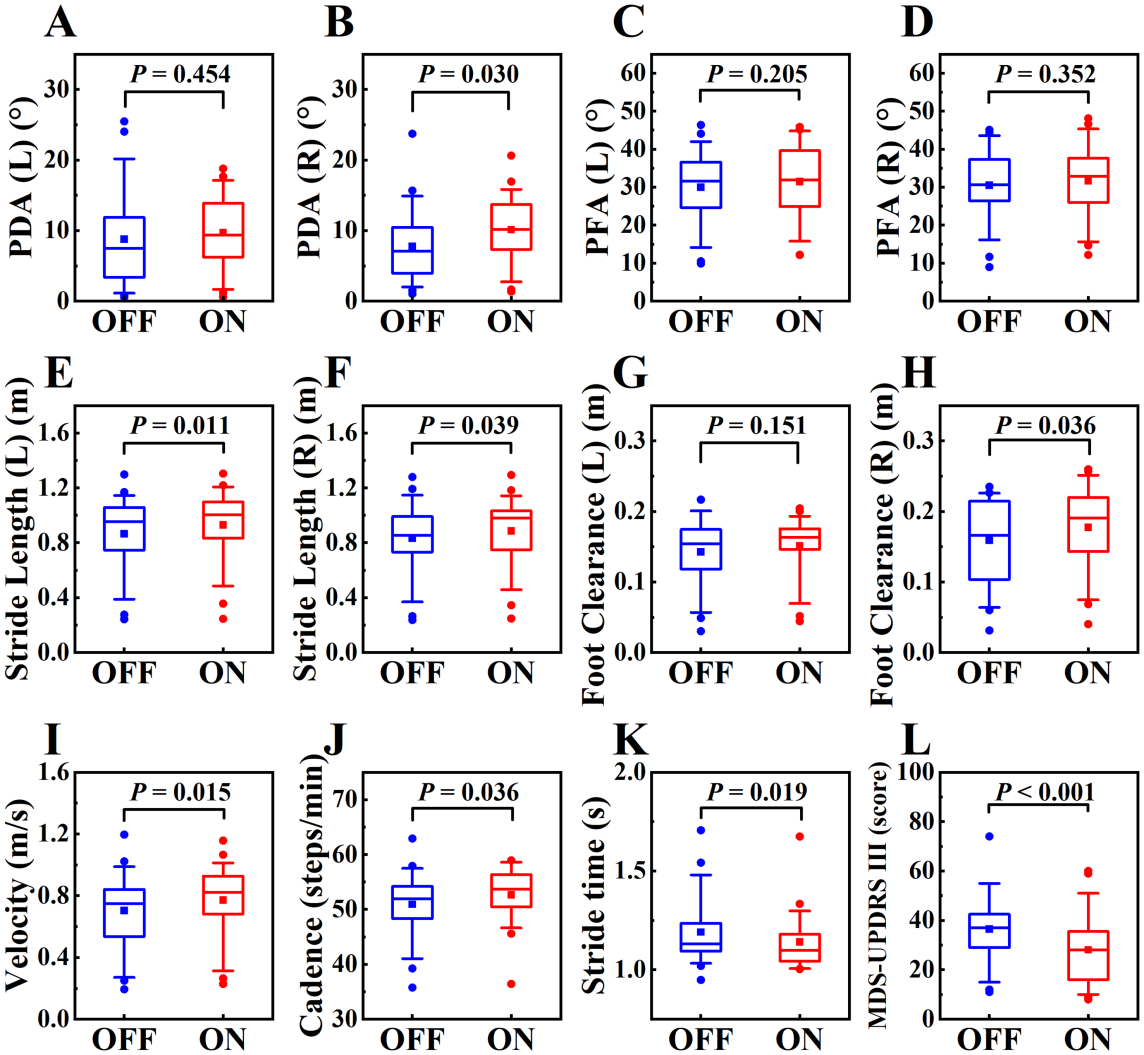
The boxplot of gait parameters and MDS-UPDRS III score in OFF and ON states in PD patients, labeled **(A–L)**, including PDA (left, right), PFA (left, right), stride length (left, right), foot clearance (left, right), velocity, cadence, stride time, MDS-UPDRS III score. The schematic diagram of the boxplot is the same as Figure 4.

We also explored the effect of levodopa on gait parameters. The differences between OFF and ON states were shown in Table 6 and Figure 6, MDS-UPDRS III score was decreased in the ON state, PDA (right), stride length (left, right), foot clearance (right), velocity, cadence were increased in the ON state, stride time was decreased in the ON state, and the differences were statistically significant (*p* < 0.05), highlighting the therapeutic effects of levodopa on gait parameters and motor symptoms in PD patients. In our study, only the PDA and foot clearance on the right side had statistically significant difference, we speculated that it might be related to the unilateral onset of PD. Among the 20 patients, 3 had left side symptoms, 6 had right side symptoms, and 11 had bilateral symptoms, which might be related to more severe symptoms on the right side.

**Table 6:** Comparison of gait parameters before and after taking levodopa in PD patients.

Variable	OFF	ON	*P* value
MDS-UPDRS III (score)	36.55 ± 15.31	28.10 ± 14.92	**< 0.001**
PDA (L) (°)	7.45 (3.26, 12.26)	9.69 ± 5.58	0.454
PDA (R) (°)	7.07 (3.73, 10.55)	10.10 ± 4.97	**0.030**
PFA (L) (°)	29.94 ± 9.98	31.46 ± 10.16	0.205
PFA (R) (°)	30.50 ± 9.73	31.63 ± 9.95	0.352
Stride Length (L) (m)	0.87 ± 0.28	1.00 (0.83, 1.10)	**0.011**
Stride Length (R) (m)	0.83 ± 0.27	0.89 ± 0.26	**0.039**
Foot Clearance (L) (m)	0.14 ± 0.05	0.16 (0.14, 0.18)	0.151
Foot Clearance (R) (m)	0.17 (0.10, 0.21)	0.18 ± 0.06	**0.036**
Velocity (m/s)	0.70 ± 0.26	0.82 (0.66, 0.93)	**0.015**
Cadence (steps/min)	50.92 ± 6.31	53.70 (50.41, 56.43)	**0.036**
Stirde time (s)	1.13 (1.09, 1.24)	1.10 (1.04, 1.18)	**0.019**

OFF, the state in which a patient has been without using dopamine agonists for at least 72 h and without using any anti-Parkinson drugs for at least 12 h. ON, the state 2 h after taking levodopa. *p* values in bold indicate statistical significance (*p* < 0.05). *p* value < 0.05 should be shown in bold.

### 3.5 Gait parameters predict MDS-UPDRS III score

It could be seen that the model had the strongest explanatory ability to the non-tremor part score (R^2^ = 0. 775) from the evaluation indexes shown in Table 7, the MDS-UPDRS III score could also be interpreted by the model (R^2^ = 0. 675), and the model was weak in interpreting the tremor part score (R^2^ = 0. 138).

**Table 7:** Evaluation indexes of regression prediction model.

Evaluation metrics prediction model	R^2^	MAE	MAPE
MDS-UPDRS III score	0.675	3.725	0.145
Tremor part score	0.138	2.082	0.829
Non-tremor part score	0.775	2.550	0.087

R^2^, R-squared; MAE, Mean Absolute Error; MAPE, Mean Absolute Percentage Error.

**Figure 7:**
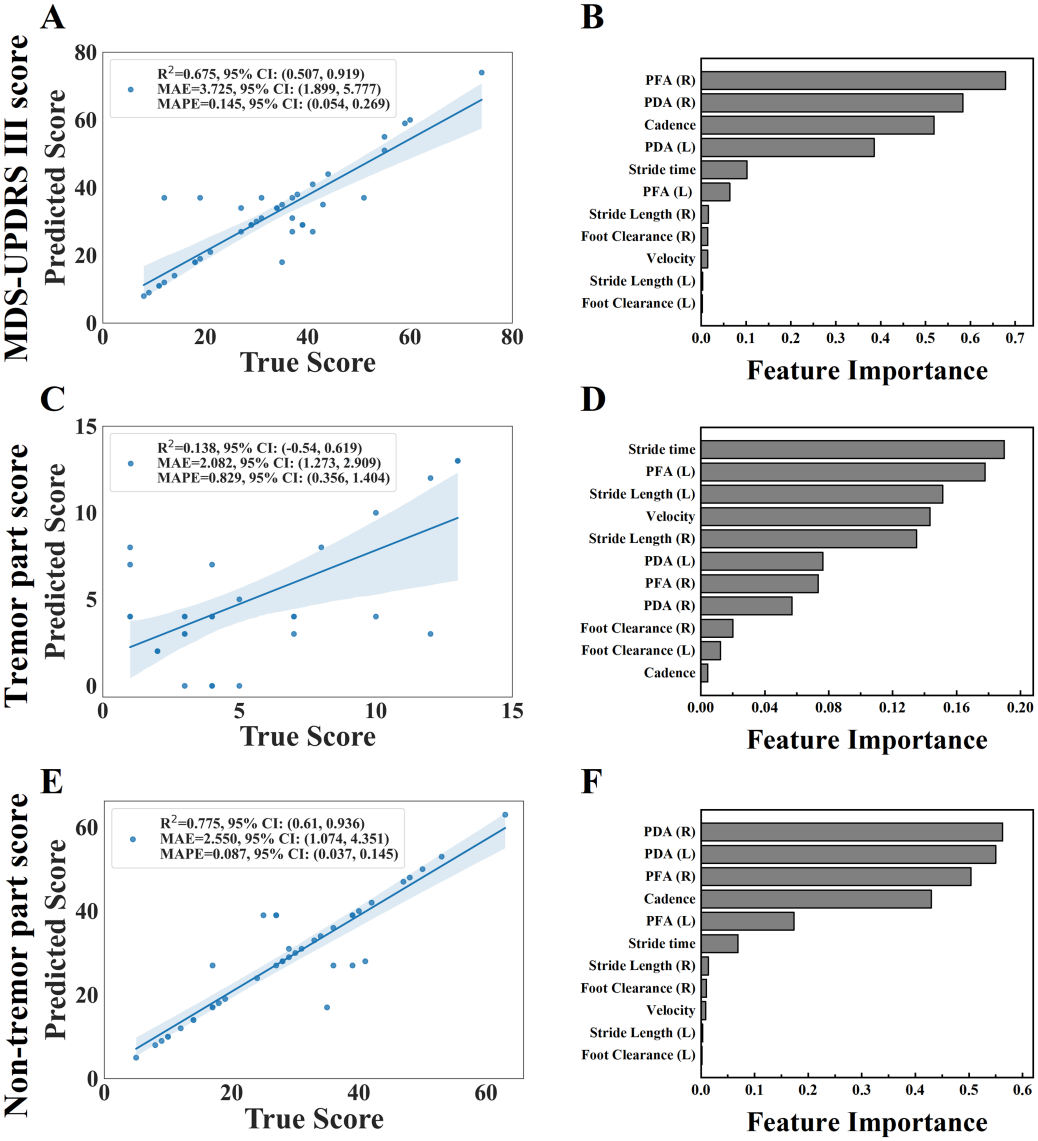
The scatter plot of the relationship between the predicted and true values of the three regression models were shown on plots **(A,C,E)**, including MDS-UPDRS III score, tremor part score and non-tremor part score. (the horizontal axis of the scatter plot represents the true value of the model and the vertical axis represents the predicted value). The feature importance were shown on plots **(B,D,F)**.

**Figure 8:**
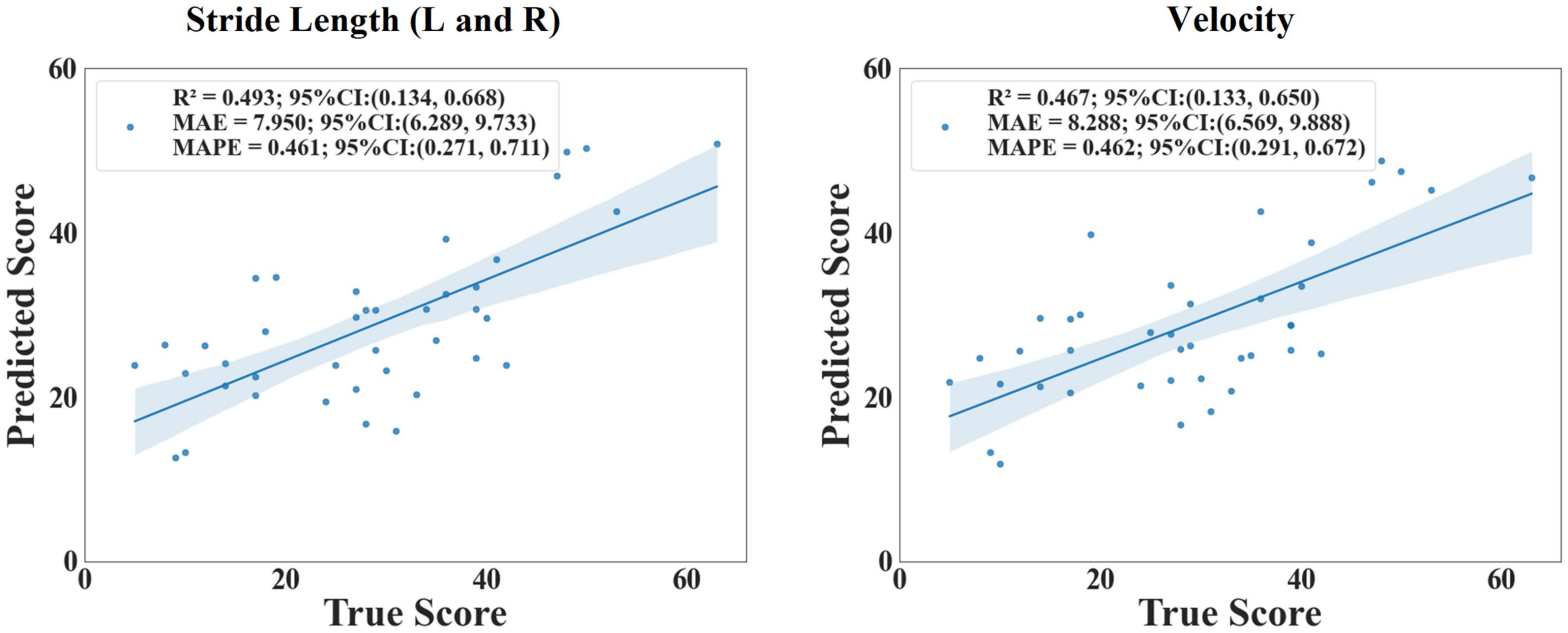
The scatter plot of the relationship between the predicted and true values of stride length and velocity.

The scatter plot (Figure 7) showed the relationship between the predicted value and the true value of the regression models. From the plot, it could be seen that the model for predicting MDS-UPDRS III score and non-tremor part score performed better. At the same time, we constructed a baseline model using only stride length and velocity to predict MDS-UPDRS sub-scores. These models yielded lower R^2^ values (e.g., stride length (left and right) model: R^2^ = 0.493 for non-tremor part score, velocity-only model: R^2^ = 0.467 for non-tremor part score), confirming the predictive value of incorporating multiple gait features. (Figure 8).

We further analyzed the feature importance of the gait parameters, feature importance was evaluated based on the values of the regression coefficients, the magnitude of each standardized coefficient reflects the strength of its association with the outcome (Figure 7). The importance degree of MDS-UPDRS III score was in the following order: PFA (right), PDA (right), cadence, PDA (left), stride time, PFA (left), stride length (right), foot clearance (right), velocity, stride length (left), foot clearance (left); the importance degree of non-tremor part score was in the following order: PDA (right), PDA (left), PFA (right), cadence, PFA (left), stride time, stride length (right), foot clearance (right), velocity, stride length (left), foot clearance (left). We could see that PDA and PFA contribute most to MDS-UPDRS III score and non-tremor part score. Each standardized coefficient of tremor part score was very small, we did not analyze it.

## 4 Discussion and conclusion

Gait impairment in PD is often characterized by short steps and a shuffling gait resulting in an increased risk of falling. It plays an important role in PD patients, affecting the quality of life, limiting the independence and activities of daily life (19). In order to determine the severity of gait disorders, early and objectively gait assessment is critical (20, 21). In this study, the gait parameters of participants were objectively and quantitatively assessed by wearable sensors. The demographic variables (age, gender, height and weight) were comparable between PD patients and healthy controls. Most gait parameters (recorded in OFF state) in PD patients were significantly different from those in healthy controls. Stride length (left, right) and velocity were significantly decreased in PD patients. However, there was no significant difference between the two groups of cadence and stride time, that is, the observed changes of gait parameters validated the known clinical characteristics of short, shuffling and slow gait disorders in PD patients, which was consistent with the existing research. Mondal et al. (22) showed that the mean velocity in PD patients was 0.74 m/s and the mean stride length was 0.86 m, while the mean velocity was 0.99 m/s and the mean stride length was 1.15 m in age-matched healthy controls. The velocity was slower and the stride length was shorter in PD patients. These changes reflected the characteristics of “bradykinesia” in PD patients. It should be noted that the main cause of bradykinesia in PD patients is short steps rather than slow cadence (22). We also explored the correlation between gait parameters and MDS-UPDRS III score. The results showed that there was a negative correlation between velocity, stride length, foot clearance, PFA and MDS-UPDRS III score, indicated that the more severe the motor symptoms of PD patients, the higher degree of gait disorders.

Our study also assessed the responsiveness of gait parameters to levodopa, and studied the relationship between gait parameters and clinical improvement in OFF and ON states. We observed an increase in velocity, stride length and cadence in PD patients after taking levodopa. This was slightly different from previous studies on the effect of levodopa on gait in PD. Most previous studies showed that the temporal parameters related to gait rhythm were resistant to levodopa, however, the parameters requiring expenditure of energy (velocity and stride length) were sensitive to levodopa (22–24). It was speculated that the improvement of gait was similar to the improvement of bradykinesia, gait parameters which related to movement amplitude and speed could benefit from dopaminergic treatment (25), while the levodopa-resistant gait parameters might be regulated by non-dopaminergic circuit (26). However, our study found that levodopa could improve the cadence and stride time, we speculated that the reason might be that the faster velocity could increase cadence and decrease stride time. Fukuchi *et al*. found that cadence was decreased when the subjects walked slowly, and cadence was increased with the increase of velocity in a meta-analysis on the influence of velocity on gait parameters in healthy subjects (27). A research by Curtze et al. (13) about the effect of levodopa on gait parameters in PD patients also showed that levodopa could increase cadence and decrease stride time.

Our study also observed that the PDA (left, right) and the PFA (left, right) in PD patients were all smaller than that in healthy controls, which indicated that PD patients inclined to have a flat-footed gait pattern during walking. However, our results were slightly different from those of previous studies. Johannes *et al*. found that the PDA of PD patients was decreased, but the PFA was not (28). Our study also found that only the right PDA was increased after taking levodopa, but the left PDA and the PFA were not. We speculated that the dorsiflexor muscles were more likely to be affected by levodopa, as for only the right PDA was increased, which might be related to the more severe motor symptoms of the right side. This consistented with the results of previous studies, levodopa could only increase PDA to a certain extent but not PFA (29). The degree of the PDA and the PFA is affected by muscles, and the lateral gastrocnemius muscle (LG) is the flexor plantaris muscle of the ankle joint, the tibialis anterior muscle (TA) is the dorsal flexor muscle of the ankle joint (30). Dopaminergic medicine can increase the activity of distal lower limb muscles, especially TA (31).

PD is often divided into two types according to motor symptoms: postural instability gait difficulty (PIGD) and tremor dominant (TD) subtypes (32). Previous studies have shown that gait disturbances are more prominent in the PIGD group compared to the TD group (33, 34). Vieregge et al. (35) indicated that gait was correlated with the bradykinesia and axial motor symptom, but not with the tremor symptom. Safarpour et al. (36) used gait parameters to predict MDS-UPDRS III score, correlation analyses were conducted between predicted score and the total MDS-UPDRS III score, MDS-UPDRS rigidity subscore, MDS-UPDRS PIGD subscore. The correlation coefficients and *p* values were as follows: *r* = 0.48, *p* = 0.0069; *r* = 0.49, *p* = 0.0059; and *r* = 0.61, *p* = 0.0059. This indicated that gait prediction performed better for the rigidity subscore and PIGD subscore than for the total score, especially for the PIGD subscore, but the study did not mention the correlation between gait parameters with the tremor subscore. Rehman et al. (37) used gait parameters to predict MDS-UPDRS III score by deep learning method and found a strong correlation (*r* = 0.82, *p* < 0.001) between predicted and actual scores, absolute agreement was good (Intraclass correlation (2,1) = 0.76, *p* < 0.001), but they did not further analyze the score by breaking it down into subscores.

To further explore the predictive value of objective gait parameters on MDS-UPDRS III score, we constructed three predictive models with output labels: MDS-UPDRS III score, tremor part score, non-tremor part score. From the value of R^2^, the model could explain the MDS-UPDRS III score and the non-tremor part score better, but performed poorly in predicting the tremor part score, this indicated limited predictive capability for the tremor part score. From the value of MAE, the MAE of the model for the tremor part score was the smallest, followed by the non-tremor part score and finally the MDS-UPDRS III score, the main reason was that the total score of the three parts were unbalanced, and the score of the tremor part was small, which leaded to the small error value. From the value of MAPE, the prediction accuracy of the model for the non-tremor part score was the best, followed by the MDS-UPDRS III score and the last was the tremor part score, which indicated that the model could explain and predict the MDS-UPDRS III score and the non-tremor part score well, and the non-tremor part score was better than the MDS-UPDRS III score. We speculate that whether the study combine gait assessment and tremor assessment will predict the MDS-UPDRS III score more accurately and objectively in the future?

Meanwhile, from the perspective of technical support, in order to improve robustness in data-scarce settings, recent studies have explored the integration of interpretable machine learning with generative AI techniques - such as conditional generative adversarial networks and synthetic data augmentation which have shown potential to enhance model generalizability while maintaining clinical relevance and transparency, particularly in rare disease applications (38). Future work in gait analysis for neurodegenerative diseases may benefit from incorporating such approaches, alongside prospective and multicenter validation strategies.

## 5 Limitation

There were some limitations in the current study. The first limitation was that we only included PD patients who were able to independently walk 10 meters or more, the MDS-UPDRS III score was low, so our results might only be applied to mild PD patients. Additionally, the relatively small sample size and single-center recruitment may constrain the generalizability of the findings. Last, the predictive models were trained and tested without external validation and lack of independent test set, this methodological constraint raised concerns regarding potential overfitting and limited the current conclusions on model performance. To ensure clinical utility and translational relevance, future studies should incorporate multicenter cohorts and prospective validation strategies.

## Data Availability

The raw data supporting the conclusions of this article will be made available by the authors, without undue reservation.
